# Screening for HIV-Associated Neurocognitive Disorder (HAND) in Adults Aged 50 and Over Attending a Government HIV Clinic in Kilimanjaro, Tanzania. Comparison of the International HIV Dementia Scale (IHDS) and IDEA Six Item Dementia Screen

**DOI:** 10.1007/s10461-020-02998-9

**Published:** 2020-09-01

**Authors:** Johanna Kellett-Wright, Aidan Flatt, Patrick Eaton, Sarah Urasa, William Howlett, Marieke Dekker, Aloyce Kisoli, Ashanti Duijinmaijer, Jessica Thornton, Judith McCartney, Vanessa Yarwood, Charley Irwin, Elizabeta Mukaetova-ladinska, Rufus Akinyemi, Bingileki Lwezuala, William K. Gray, Richard W. Walker, Catherine L. Dotchin, Philip Makupa, Stella-Maria Paddick

**Affiliations:** 1 Newcastle University, Newcastle upon Tyne, UK; 2 Kilimanjaro Christian Medical University College, Moshi, Kilimanjaro Tanzania; 3 Haydom District Hospital, Manyara, Tanzania; 4 The London School of Hygiene & Tropical Medicine, London, UK; 5 Department of Neuroscience, Behaviour and Psychology, University of Leicester, Leicester, UK; 6 The Evington Centre, Leicestershire Partnership NHS Trust, Leicester, UK; 7 University of Ibadan, Oyo State, Nigeria; 8 Mawenzi Regional Referral Hospital, Moshi, Kilimanjaro Tanzania; 9 Northumbria Healthcare NHS Foundation Trust, North Tyneside General Hospital, North Shields, UK; 10 Gateshead Health NHS Foundation Trust, Gateshead, UK

**Keywords:** HIV, Older adults, Sub-Saharan Africa, Cognitive impairment, Screening, IHDS

## Abstract

Validated screening tools for HIV-associated neurocognitive disorders (HAND) are lacking for the newly emergent ageing population of people living with HIV (PLWH) in sub-Saharan Africa (SSA). We aimed to validate and compare diagnostic accuracy of two cognitive screening tools, the International HIV dementia scale (IHDS), and the Identification and Interventions for Dementia in Elderly Africans (IDEA) screen, for identification of HAND in older PLWH in Tanzania. A systematic sample of 253 PLWH aged ≥ 50 attending a Government clinic in Tanzania were screened with the IHDS and IDEA. HAND were diagnosed by consensus American Academy of Neurology (AAN) criteria based on detailed clinical neuropsychological assessment. Strict blinding was maintained between screening and clinical evaluation. Both tools had limited diagnostic accuracy for HAND (area under the receiver operating characteristic (AUROC) curve 0.639–0.667 IHDS, 0.647–0.713 IDEA), which was highly-prevalent (47.0%). Accurate HAND screening tools for older PLWH in SSA are needed.

**Electronic supplementary material:**

The online version of this article (10.1007/s10461-020-02998-9) contains supplementary material, which is available to authorized users.

## Resumen

Faltan pruebas cognitivas válidas para los trastornos neurocognitivos asociados al VIH (según sus siglas en inglés, HIV-Associated Neurocognitive Disorder (HAND) en la población emergente de personas mayores que viven con el VIH en el África subsahariana. Nuestro objetivo era validar y comparar la precisión diagnóstica de dos pruebas cognitivas, la escala internacional de demencia por VIH (según sus siglas en ingles International HIV dementia scale (IHDS) y la prueba ‘IDEA’, para el cribado de trastornos neurocognitivos asociados al VIH (HAND) en personas mayores viviendo con VIH en Tanzania. Una muestra sistemática de 253 personas de ≥50 años que asistieron a una clínica gubernamental en Tanzania se examinó con el IHDS y la IDEA. HAND fueron diagnosticados por consenso según los criterios de la Academia Americana de Neurología (AAN) basados en una detallada evaluación neuropsicológica y clínica. Las fases de cribado y de evaluación clínica se realizaron de forma independiente y a ciegas. Ambas herramientas tenían una precisión de diagnóstico limitada para HAND (área bajo la característica de funcionamiento del receptor (AUROC) curva 0.639 – 0.667 IHDS, 0.647-0.713 IDEA). HAND era altamente frecuente (47%). Se necesitan pruebas cognitivas por cribado de deterioro cognitivo en personas mayores con VIH en el África subsahariana.

## Introduction

HIV-associated neurocognitive disorders (HAND) are highly prevalent in people living with HIV (PLWH) worldwide and are associated with substantial morbidity and disability [1–4]. Older PLWH appear to be at highest risk, with up to 50% affected in high-income countries (HICs) [5].

HAND are poorly understood, but evidenced contributory mechanisms include opportunistic infections of the central nervous system, direct neurotoxic and inflammatory effects of the HIV virus, and neurotoxic effect of combination antiretroviral therapy (cART) [6, 7].

Prior to widespread availability of cART, a rapidly-progressive subcortical dementia (’AIDS dementia complex’, now termed HIV-associated dementia (HAD)), was commonly observed in advanced HIV/ AIDS. Evidence from both HICs and sub-Saharan Africa (SSA) suggests that cART has resulted in a milder but more prevalent, broader, clinical phenotype of cognitive deficit in HIV [8–10].

Diagnostic criteria for HAND have been updated to define this broader, milder spectrum of disorders on a continuum encompassing Asymptomatic Neurocognitive Impairment (ANI), Mild Neurocognitive Disorder (MND) and HIV-associated Dementia (HAD), depending on severity of observed cognitive deficit and the presence, or absence of functional impairment [11].

HAND are likely to disproportionately affect SSA, where more than two thirds of new HIV infections occur, but rapidly increasing cART coverage is rapidly increasing life expectancy in PLWH [12]. As a result, HIV prevalence is increasing and, as in HICs, the PLWH population is ageing rapidly [13]. PLWH aged ≥ 50 in SSA are predicted to triple by 2040, increasing from one in seven to one in four of the total [14]. HAND are likely to increase rapidly in prevalence in this newly-ageing population.

Despite this, the prevalence of HAND in older cART-treated PLWH in SSA is not currently known. Existing SSA prevalence estimates for HAND are of younger populations, frequently excluding those aged ≥ 45  [15]. Moreover, these existing studies utilise differing methodologies and diagnostic criteria for HAND, and combine cART-treated and untreated PLWH. It follows that these prevalence estimates vary markedly, with rates of 15.6–80.0% reported [1]. Current epidemiological studies of HAND in SSA therefore do not reflect this cART-treated newly-emergent and rapidly increasing ageing population of PLWH.

An important issue is that, in this resource-limited setting, many epidemiological studies rely on HAND screening tools for prevalence estimates [15]. Formal diagnostic criteria for HAND require demonstration of deficit in two neurocognitive domains, usually evaluated using a comprehensive neuropsychological test battery [11] alongside clinical assessment by an experienced clinician to exclude other potential causes of poor cognitive performance. Assessment of this type is often impractical to complete in routine clinical practice, particularly in low-resource settings such as SSA, where neurology, psychiatry and geriatric medicine specialists are few [16, 17]. Accurate screening measures are therefore needed for both clinical practice and research.

Screening for mental disorders and HAND is recommended by HIV guidelines in HICs and the WHO. However there is no current consensus on the tools to be used [18, 19], or indeed the individuals who should be targeted for screening since untargeted screening may overestimate HAND prevalence by approximately 20% due to overlap of milder forms of HAND with other comorbid conditions [20].

The International HIV dementia Scale (IHDS) is one of the most commonly used HAND-specific screening tools worldwide [21, 22]. The IHDS was initially developed in the USA and Uganda to be cross-culturally applicable and useful in lower-literacy settings in SSA in comparison to the previously established HIV dementia scale (HDS) [21] widely used in the USA. Moreover, recent systematic reviews suggest overall diagnostic accuracy may be lower than in the original validation studies, possibly because of the changing profile of HAND with cART [22, 23]. It is important to note that the IHDS was developed for, and validated against, diagnostic criteria for ‘HIV-associated dementia’, whereas increasingly even in SSA, the widespread availability of cART has resulted in the milder and broader concept of HAND being well recognised. The IHDS has not previously been validated in older PLWH in SSA, and the accuracy of this screening measure in the newly emergent population of PLWH ageing on cART in SSA is not known. It is likely however, that the profile of cognitive impairments occurring in this population may differ to that seen in HICs due to differing demographics and comorbidities [24].

The clinical phenotype of HAND appears to differ in older versus younger PLWH and may be confounded by neurodegenerative processes. Accelerated ageing and cerebral amyloid deposition as seen in Alzheimer’s disease (AD) are hypothesised to occur in older PLWH [25]. AD typically presents with a cortical pattern of language and memory deficits, observed in more recent studies of older PLWH in addition to the classical subcortical deficits seen in HIV-associated dementia and the newer categorisation of HAND, where executive function and motor speed would more commonly affected [26]. Atherosclerosis and changes in lipid metabolism leading to cerebrovascular disease may also result in vascular cognitive impairment disproportionately affecting older PLWH [24, 27]. Older PLWH with HAND may therefore present with a differing profile of cognitive impairment, more typical of neurodegenerative dementias.

The IDEA screen is a brief low-literacy cognitive screen (Online Appendix 1) previously-validated for delirium and neurodegenerative dementias in hospital and community samples aged ≥ 65 in Tanzania and Nigeria [28–30]. Since the IDEA covers a broad range of cognitive domains [28], and was locally validated, we hypothesised it might be a useful alternative screen for cognitive impairment in older PLWH.

We aimed to conduct a blinded validation study of the accuracy of the IHDS and IDEA screen for HAND, diagnosed by current American Academy of Neurology (AAN) criteria in individuals aged ≥ 50 under long term follow-up at a standard Government free-of-charge HIV clinic in Tanzania. Additionally, we aimed to compare diagnostic accuracy of both measures to determine which might be the most clinically useful in routine HAND screening of older PLWH in SSA.

## Methods

### Ethical Consideration

The Tanzanian National Institute for Medical Research, and Kilimanjaro Christian Medical University College Ethical Review Committee approved the study. Trained study nurses obtained written informed consent. Individuals unable to read and write indicated consent via thumbprint after a verbal explanation of the purpose and implications of the study.

Where capacity to give valid consent was in doubt due to cognitive deficit, written assent was obtained from a close relative. Incentives were not paid, but appropriate refreshments provided and additional transport costs reimbursed. A locally-agreed protocol for appropriate onward referral of conditions identified by study clinicians was a key element of study design.

### Participants and Setting

This study took place between March and June 2016 in the HIV Care and Treatment Centre (CTC) of Mawenzi Regional Referral Hospital (MRRH) in Kilimanjaro, Tanzania. National HIV prevalence is currently 3.9% (95% CI 3.6–4.3%) [31]. MRRH is a Government-funded facility and as a cART pioneer site has provided free-of-charge HIV treatment and follow-up for over 15 years resulting in a long term treated cohort of PLWH. In 2016, 820 of 1352 registered patients (25.4%) were aged ≥ 50.

**Fig. 1:**
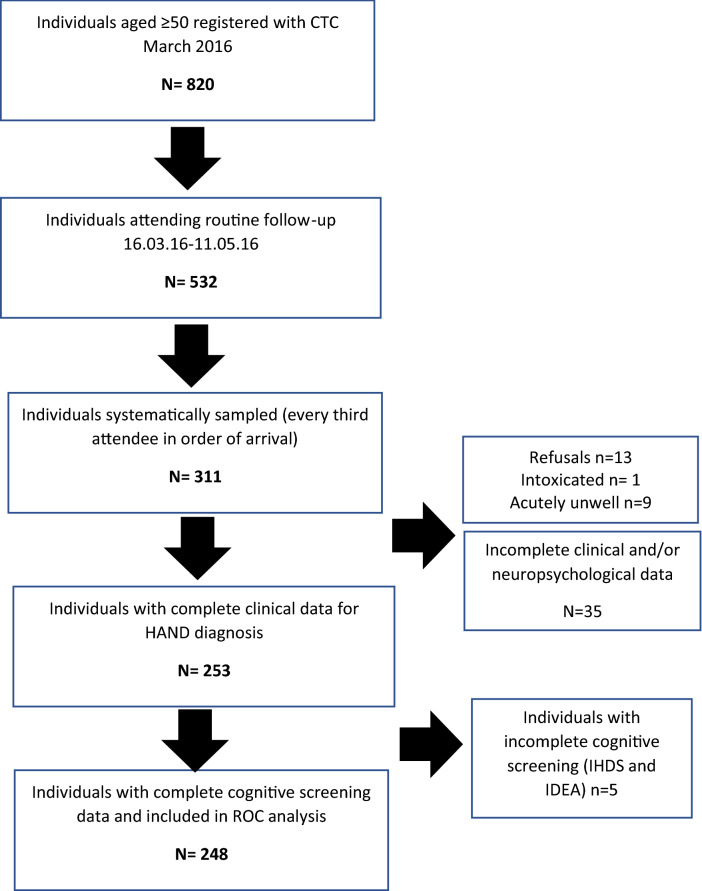
Study flowchart and exclusions

A systematic random sample of PLWH aged ≥ 50 were recruited in order of arrival to the clinic for routine follow-up appointments (see Fig. 1). All those consenting were eligible for inclusion except in cases of acute illness and/or urgently necessary medical treatment or lack of capacity to consent in the absence of a close relative to give written assent. A required sample size of 245 was calculated based on a predicted prevalence of ‘HIV dementia’ by 1991 criteria [32] of ≥ 20% approximating to current combined MND/HAD categories (s-HAND) on current criteria [11] with sensitivity and sensitivity of 85%, power of 80% and 95% confidence level. This measure was selected based on the largest number of existing studies.

### Neurocognitive Assessment for HAND

All individuals underwent a comprehensive neurocognitive assessment based on current American Academy of Neurology (AAN) criteria for HAND [11] blinded to outcome of cognitive screening.

### Neuropsychological Assessment for HAND

Assessment included a detailed neuropsychological battery based upon that used in the original WHO cross-national studies of HIV-associated dementia (HAD) [33, 34]. This was locally normed for age and education with control subjects attending other MRRH chronic disease clinics. Additional tests of cortical function and low-literacy adaptations were based upon previous validation studies and clinical experience of cognitive assessment instruments in Tanzania by our team [35, 36]. Locally-normed tests in the battery included: motor speed (grooved pegboard test, 10 m timed walk), executive function (color trails 1 and 2), constructional praxis (stick design test), attention and working memory (digit span forwards and backwards), learning and memory (Auditory Verbal Learning Test (AVLT), command comprehension, orientation and categorical verbal fluency (market items). The battery was administered by specialist nurses or occupational therapists fluent in both Swahili and English after a training and harmonization period and scored by observers (J K-W, AF, PE).

### Clinical Assessment

Local translations of the Mini International Neuropsychiatric Interview (MINI) [37] and 15-item Geriatric depression scale (GDS) [38] administered by a doctorate-level-specialist nurse screened potentially-confounding psychiatric disorders. Subjective cognitive and neurological symptoms were screened through self-report.

Clinical assessment included structured mental state examination, and bedside cognitive and neurological assessment with a research doctor (CI, JM, JT, VY). Bedside cognitive assessment incorporated major cognitive domains (orientation, registration and delayed recall, attention, receptive and expressive language, praxis and frontal lobe function,using Luria’s three hand position test) to confirm neuropsychological test findings and assist in exclusion of delirium using the Confusion Assessment Method (CAM), previously validated locally [39]. Functional impairment and history were confirmed through structured collateral history from a close informant (where necessary by telephone), including a locally-validated instrumental activities of daily living (IADL) scale [40]. Detailed clinical case notes were prepared, and a provisional diagnoses assigned following multi-disciplinary discussion involving all clinicians involved in assessment. Complete assessment lasted up to three hours.

### HAND Diagnosis

HAND diagnosis by 2007 AAN criteria was based on detailed consensus panel discussion considering all available clinical information by specialists in old age psychiatry and neurology (EM-L, S-MP, RA). Diagnoses of other dementias, delirium and other significant mental illness, where present, were based on DSM-5 criteria [41].

### Cognitive Screening

Cognitive screening was completed prior to additional assessments and screeners remained blinded to the outcome of additional cognitive assessments. Screening tools were administered by senior research nurses and occupational therapists with previous experience of similar studies, following a week-long harmonisation and training process.

### IDEA Six-Item Screen

The IDEA screen is a locally-developed low-literacy cognitive screen previously-validated for non-HIV neurodegenerative dementias and delirium in community and hospital settings in Tanzania, Nigeria, and Malaysia [29, 30, 39, 40, 42]. It includes delayed recall, orientation, two measures of frontal lobe function, verbal fluency and abstract reasoning, praxis and long-term memory without literacy-dependent items. The maximum score is 15.0 with higher scores indicating better performance. Scores < 10/15 are considered indicative of cognitive impairment and ≤ 7/15 is the previously validated cut-off for major cognitive impairment (dementia and delirium) in SSA.

### International HIV Dementia Scale (IHDS)

The International HIV dementia scale (IHDS) was designed for SSA and includes a brief screen of registration, verbal recall, motor and psychomotor speed without literacy-dependent tests. The maximum score is 12.0 with higher score indicative of better performance. The original validation study suggested a cut-off of ≤ 10.0 for HIV dementia (sensitivity 80% and specificity 55%) in an urban cohort of PLWH [21] in Uganda.

### Blinding

The clinicians administering neuropsychological and clinical assessments were blinded to screening results by a coordinator who ensured that blinding was maintained. Screening and neuropsychological assessments took place in separate rooms and screening tests were filed on completion separate from all other study documentation.

### Statistical Methods

Data analysis was supported by IBM SPSS (version 23; IBM, Armonk, NY, USA). The performance of the screening tools was investigated using area under the receiver operating characteristic (AUROC) curve analysis alongside sensitivity, specificity, positive predictive value (PPV) and negative predictive value (NPV). Optimal cut-off values were determined using the ROC analysed at 0.5 increments, and considered a positive screen result if less than or equal to the stated value. Diagnostic accuracy was analysed for HAND, symptomatic HAND (s-HAND, MND/HAD) and HAD as these data would have clinical utility. To determine diagnostic accuracy for HAND, ANI/MND/HAD were coded 1, and all others (including other cognitive diagnoses) coded 0. Similarly for s-HAND, MND/HAD were coded 1, and all others 0. The AUROC was selected as a good global measure of the ability of a scale to detect presence of given criteria. Other measures of diagnostic accuracy could have been used, however the AUROC is well understood by clinicians [43] and facilitated comparison to previous clinical studies. We felt this to be most appropriate given the clinical focus of this study.

Standard descriptive statistics (e.g. mean, median, standard deviation (SD), interquartile range (IQR) and frequency) were used depending on the level and distribution of the data. Statistical significance was set at 5% and two-tailed tests used throughout.

## Results

Complete clinical assessment data, allowing formal consensus HAND diagnosis, were available for 253 individuals from 311 sampled. Median age was 57.0 years (range 50.0–79.0, IQR 53.0–61.5) and 183 (72.3%) were female. Most were in employment (86.2%) indicating high functional status. Almost all (95.5%) were receiving cART, according to pre-2017Tanzanian guidelines (cART at CD4 < 350 or WHO HIV stage 4) with a median of 7.1 years since diagnosis. Only 9.9% were receiving second-line treatment and median CD4 count was good (516), although HIV viral load testing was locally unavailable in 2016.

Three individuals failed to complete the IDEA screen and 5 the IHDS therefore diagnostic accuracy was calculated for the 248 with complete data. All exclusions are detailed in Fig. 1.

In the full cohort (n = 253), HAND were identified in 47.0% (n = 119) of whom 64 people (25.3%) met criteria for Asymptomatic Neurocognitive Impairment (ANI), 46 (18.2%) Mild Neurocognitive Disorder (MND) and 9 (3.6%) HIV-associated Dementia (HAD). S-HAND was present in 55 people (21.7%). Characteristics of the cohort and other psychiatric diagnoses with potential to result in cognitive impairment are presented in Table 1.

**Table 1:** Characteristics of the baseline cohort (n = 253)

Median age	57.0 (range 50.0 -79.0)
Gender—female (n, %)	183 (72.3%)
Years since HIV diagnosis (Mn, SD) Missing = 4	7.1 (3.3) (range 0.7- 23.9)
Current cART regimen	
First-line^a^	n = 211 (83.3%)
Second-line^b^	n = 25 (9.9%)
On cART^c^	95.5%
Missing = 17	
Most recent CD4 (Mn, SD)	516.45 (255.19) (Range 98.00–1719.00)
Highest educational level	
Less than 1 year or none	29 (11.7%)
Some primary school	58 (23.4%)
Completed primary school	106 (42.7%)
Some secondary school	38 (15.3%)
Completed secondary school	13 (5.2%)
Tertiary education	4 (1.6%)
5 missing values	
Employment	
Continued employment	n = 218 (86.2%)
Prevalence of HAND	
ANI	n = 64 (25.3%, 95% CI 19.9–30.7%)
MND	n = 46 (18.2%, 95% CI 13.4–22.9%)
HAD	n = 9 (3.6%, 95% CI 1.3–5.8%)
Additional and secondary diagnoses	
Vascular cognitive impairment	10 cases
Alcohol-related cognitive impairment	7 cases (2 with previous head injury, one likely learning disability)
Possible delirium/post delirium cognitive impairment	5 cases
Other psychiatric diagnoses	
Major depression DSM-IV	42 Cases (one with psychosis) (16.6%)
Schizophrenia	1 case
Substance abuse/dependence	None
Alcohol dependence	None
Anxiety disorder	2 cases

^a^First-line regimens (NRTI × 2 + NNRTI efavirenz/nevirapine) 1 g-A (TDF, 3TC, EFV) 1b-A (AZT, 3TC, NVP/ABC,3TC, LPV/r), 1c-A(AZT, 3TC, EFV), 1e-A (TDF,FTC, EFV) 1f-A (TDF,FTC,NVP), 1 h-A (TDF, 3TC, NVP), 1 k-A (ABC, 3TC, EFV), 1 m-A (ABC, 3TC, NVP), 1a-A (d4T, 3TC, NVP/d4T, 3TC, EFV), 1x-A (other 1st line unspecified)

^b^Second-line regimens (NRTI × 2 + Protease Inhibitor (PI) 2f-A (TDF, FTC, LPV/r), 2 h-A (TDF,FTC,ATV/r), 2 s-A (AZT,3TC,ATC/r), 2 g-A (ABC,3TC,LPV/r), 2e-A (TDF, 3TC, LPV/r), 2 k-A (ABC/3TC, ATV/r), 2 m-A (TDF, 3TC, ATV/r), 2n-A (AZT, 3TC, LPV/r/AZT, 3TC, EFV), 2x-A (other 2nd line unspecified)

Tanzanian guidelines at baseline study indicated cART should commence at CD4 ≤ 350 or WHO stage 4

### Diagnostic Accuracy

The diagnostic accuracy of the IHDS and IDEA screen for HAND, s-HAND and HAD at previously validated cut-off scores is presented in Table 2. Overall diagnostic accuracy was poor. AUROC ranged from 0.639–0.667 for the IHDS and 0.647–0.713 for the IDEA depending on diagnostic HAND category. Optimal cut off scores for each of the three diagnostic categories (HAND, symptomatic HAND and HAD) Table 3 were substantially higher than previously-published cut-offs for IDEA and lower for the IHDS.

**Table 2:** AUROC for the IHDS and IDEA cognitive screen and their originally validated cut-off scores

	Median score of those who had the condition	Median score of those who did not have the condition	AUROC (95% CI)	Sensitivity	Specificity	Positive predictive value	Negative predictive value
IHDS (n = 148) Cut off ≤ 10.0							
HAND (n = 118)	8.0 (6.9 to 9.0)	9.0 (7.5 to 10.1)	0.639 (0.571 to 0.708)	0.890	0.454	0.517	0.711
Symptomatic HAND (n = 54)	8.0 (6.0 to 9.0)	8.8 (7.5 to 10.0)	0.647 (0.561 to 0.733)	0.889	0.201	0.236	0.867
HAD (n = 9)	7.5 (5.0 to 8.8)	8.5 (7.0 to 10.0)	0.667 (0.484 to 0.849)	0.889	0.184	0.039	0.978
IDEA cognitive screen (n = 148) Cut off ≤ 7.0							
HAND (n = 118)	13.0 (11.0 to 14.0)	14.0 (13.0 to 15.0)	0.647 (0.579 to 0.716)	0.017	1.000	1.000	0.528
Symptomatic HAND (n = 54)	13.0 (11.0 to 13.3)	14.0 (13.0 to 15.0)	0.713 (0.638 to 0.788)	0.019	0.995	0.500	0.785
HAD (n = 9)	13.0 (9.5 to 13.5)	14.0 (12.0 to 15.0)	0.690 (0.517 to 0.863)	0.111	0.996	0.500	0.967

**Table 3:** Optimal cut-offs for the IHDS and IDEA cognitive screen

	Cut-off	Overall diagnostic accuracy (%)	Sensitivity	Specificity	Positive predictive Value	Negative predictive value
IHDS						
HAND (n = 118)	≤ 9	60.9	0.777	0.454	0.565	0.690
Symptomatic HAND (n = 54)	≤ 6.5	76.2	0.370	0.871	0.444	0.833
HAD (n = 9)	≤ 6.5	81.5	0.444	0.828	0.089	0.975
IDEA cognitive screen						
HAND (n = 118)	≤ 13	61.3	0.610	0.615	0.590	0.635
Symptomatic HAND (n = 54)	≤ 13	62.1	0.759	0.582	0.336	0.897
HAD (n = 9)	≤ 13	52.8	0.778	0.519	0.057	0.984

For both IHDS and IDEA, AUROC values and diagnostic accuracy increased for s-HAND versus all HAND, Tables 2 and 3. Specificity of the IHDS was better for s-HAND than broadly defined HAND (87.1% vs 54.4%) and sensitivity of the IDEA 75.9% for s-HAND compared to 61.0% for all HAND. PPV was low (HAND 0.517 vs s-HAND 0.236) despite relatively high prevalence (HAND 47.6% vs s-HAND 21.8%) though NPV was higher. Of all screening options evaluated, the highest screening accuracy was achieved for s-HAND using the IDEA screen at ≤ 13/15, (AUROC = 0.713, sensitivity 75.9%, specificity 58.2% but PPV remained low at 0.336).

Due to the overall poor screening performance of both tools, the predictive ability of individual IDEA screen items were examined for potentially useful measures for HAND. Results are presented in Table 4. No item performed particularly well, though categorical verbal fluency appeared moderately useful in identifying HAD. Unsurprisingly, HAND as a broad category was the hardest to identify. The matchstick item (assessing praxis) was universally poor at identifying HAND.

**Table 4:** AUROC for individual items of the IDEA cognitive screen

	Median score of those with the condition	Median score of those who did not have the condition	AUROC (95% CI)
Delayed word recall			
HAND (n = 118)	4.0 (2.0 to 5.0)	4.0 (2.0 to 5.0)	0.669 (0.601 to 0.737)
Symptomatic HAND (n = 54)	3.0 (2.0 to 4.0)	5.0 (3.0 to 5.0)	0.721 (0.643 to 0.799)
HAD (n = 9)	3.0 (2.0 to 4.5)	4.0 (3.0 to 5.0)	0.676 (0.496 to 0.855)
Immediate word recall, sum of three attempts			
HAND (n = 118)	15.0 (12.8 to 17.0)	16.0 (14.0 to 18.0)	0.637 (0.567 to 0.706)
Symptomatic HAND (n = 54)	13.0 (11.0 to 16.0)	16.0 (14.0 to 18.0)	0.717 (0.639 to 0.795)
HAD (n = 9)	13.0 (9.0 to 15.5)	15.0 (13.0 to 17.0)	0.702 (0.523 to 0.881)
Number of animals named			
HAND (n = 118)	12.0 (9.0 to 14.0)	14.0 (11.0 to 17.0)	0.623 (0.553 to 0.694)
Symptomatic HAND (n = 54)	10.0 (8.0 to 13.0)	13.0 (11.0 to 16.0)	0.702 (0.622 to 0.782)
HAD (n = 9)	8.0 (5.5 to 11.5)	13.0 (10.0 to 16.0)	0.796 (0.642 to 0.950)
Matchsticks item score			
HAND (n = 118)	3.0 (3.0 to 3.0)	3.0 (3.0 to 3.0)	0.512 (0.439 to 0.585)
Symptomatic HAND (n = 54)	3.0 (3.0 to 3.0)	3.0 (3.0 to 3.0)	0.536 (0.445 to 0.627)
HAD (n = 9)	3.0 (3.0 to 3.0)	3.0 (3.0 to 3.0)	0.531 (0.328 to 0.734)

## Discussion

This cohort had relatively well managed HIV disease and were comparable to those in HIC HIV services. The high overall HAND prevalence and pattern of impairments seen (with predominant milder HAND) also mirror those now seen in cART treated older PLWH in HICs.

The IHDS and IDEA had poor diagnostic accuracy for broadly defined HAND. Although sensitivity improved for the narrower category of s-HAND, specificity and PPV were low. In low-resource settings where a second, more detailed assessment to exclude other causes of poor performance may be impractical, use of the IHDS or IDEA to identify those with broader HAND and/or s-HAND in clinical or research contexts may have serious limitations. The high NPV observed may have utility in identifying those without HAND or s-HAND, and likely to be cognitively robust [44]. Other SSA IHDS validation studies have reported varied sensitivity (45% to 100%) and specificity (37.0% to 79.0%) [45]. Major factors likely to be affecting performance of the IHDS in this context include demographic factors (particularly age and educational background), comorbidities, heterogeneity of impairments now seen in HAND, and controversies over the validity of HAND diagnosis itself. These will be considered in turn.

Educational level and especially illiteracy are well-recognised to affect performance on cognitive screening [46] even in tests not literacy-dependent such as Luria’s three hand position test [47] (included in the IHDS). The IHDS was validated in a relatively well-educated (mean 8.7 years), younger (mean age 37) urban cohort. In comparison, our older cohort were substantially lower-educated, but literacy levels (82.8%) were high in comparison to national literacy data for adults aged ≥ 65 in Tanzania (43%) [44].

A large recent East African validation study (conducted in Kenya, Uganda and Tanzania) also identified a lower optimal IHDS cut-off of ≤ 8.0/12.0 as in our study, with 83% classified as HAD at the standard cut-off). As in our study, this lower score did not substantially improve accuracy [48]. Educational level (64–68% completed primary education, 93% literate) was only slightly higher than in our study, though adults of all ages were included and only 68.0% were cART treated.

The IDEA screen, though validated for major cognitive impairment (delirium and dementia) in older people locally and other LMIC settings, did not perform well for screening of HAND or s-HAND in this cohort of older PLWH. The major reason for this appears to have been significant ceiling effects in those with and without HAND (median 13.0/15.0). Educational level in our cohort, though low, was substantially higher than the cohorts in whom the IDEA was originally developed (rural Tanzanian elders with 2/3 illiteracy in females) and the median age [49] substantially lower.

Individual screening items within IDEA similarly did not perform well. It seems likely that these items, designed to screen for dementia in a much older and less educated population are simply too crude to have detect the more subtle impairment seen in HAND.

This issue may not exclusive to the IHDS and IDEA screen. Recent summaries of HAND screening tools worldwide (including SSA) suggest that diagnostic accuracy remains suboptimal [22, 23] for the IHDS and other tools. A large validation study conducted in the USA and South Africa with a high proportion of s-HAND concluded that the IHDS had reasonable sensitivity (68%) and specificity (86%) for s-HAND, but no test evaluated (including MOCA, MMSE, Simioni symptom questionnaire, CAT-rapid) had good performance in detection of HAND generally [48]. Similarly the MOCA-basic appears to lack clinical utility for screening cognitive decline in PLWH in SSA [50].

Current challenges in cognitive screening for HAND are likely to be due to comorbidities and heterogeneity of clinical presentation. Comorbidities frequently seen in PLWH are well recognised to both adversely impact cognition and be difficult to separate from ‘pure’ HAND. In older PLWH neurodegenerative comorbidities are increasingly recognised but difficult to separate diagnostically [51] and particularly in milder HAND. Overlap with other conditions such as vascular cognitive impairment (VCI) is increasingly recognised [52]. These changing clinical phenotypes [26] may indicate that existing diagnostic criteria no longer accurately reflect HAND presentation [53]. We did not however find screening of verbal memory or parietal function within the IDEA screen diagnostically useful, suggesting that AD-type impairments may not be the predominant presentation in our cohort.

In both HIC and LMIC settings, there is controversy regarding the benefits of screening for milder HAND [20, 54] or ANI. ANI is well-recognised to progress to more severe impairments [55]. If recognised; it may be reversible through optimisation of cART and other pharmacotherapeutic interventions [49]. Nevertheless, current guidelines do not currently advocate routine cognitive screening of asymptomatic PLWH in HIC HIV clinics [18], in part due to difficulty in accurate identification of these individuals. A solution for low-resource settings, which may reduce diagnostic and screening challenges, is to focus on s-HAND (MND/HAD). The IHDS generally appears more accurate in identification of s-HAND and was in fact developed for screening for ‘HIV-dementia’, which is similar in scope to s-HAND. In this setting however diagnostic accuracy was also suboptimal for s-HAND, with a high false positive rate.

It seems likely that broadly defined HAND by AAN criteria include deficits that are subtle and difficult to screen for with existing tools. Similar issues are well-recognised in screening for milder neurodegenerative disorders such as mild cognitive impairment (MCI), now termed minor neurocognitive disorder in DSM-5 [56] where impairments are mild, and heterogenous and of uncertain significance but the risk of progression is high. In mild vascular cognitive impairments (VCI) timed tests able to detect subtle change are prioritised alongside measures of subjective cognitive concerns (SCC). Cognitive tests for ‘dementia’ are expected to be normal despite SCC. Some initial work has taken place elsewhere in SSA exploring the efficacy of adding a measure of self-reported cognition to the IHDS and may be a useful focus for future work.

## Conclusions

Screening for HAND in older PLWH remains challenging. There are currently no validated brief screening tools with acceptable diagnostic accuracy to recommend use in routine clinical practice in older PLWH in SSA. The difficulties outlined in use of these two existing measures of HAND and neurodegenerative dementia in a typical Government service in Tanzania illustrate the challenges.

Further work should elucidate the clinical phenotype of HAND in older PLWH in SSA in order to determine the cognitive domains most affected and those at highest clinical risk to inform future works towards development of a HAND screening tool, and to identify those in greatest need of screening given the challenges seen.

## Limitations

Several limitations are acknowledged. Much existing HIC HAND literature focuses on ‘ultra-normal’ cohorts of PLWH without co-morbidity. Performance of screening tools in detecting ‘pure’ HAND will be better in these settings but these are not reflective of most clinical contexts. Our cohort was heterogenous and included individuals with other cognitive impairment in addition to, or comorbid with, HAND (see Table 1). We classified patients with clear clinical evidence of another predominant cause of cognitive impairment (VCI, alcohol, schizophrenia) as non-HAND for the purpose of analysis, but diagnosed comorbidities were highly prevalent on clinical criteria. Other comorbidities will have remained undiagnosed as large diagnostic gaps, particularly for neurological and non-communicable disease are well recognised in this setting and self-report inaccurate [57–59]. This heterogeneity may have reduced the diagnostic accuracy of the IHDS. Exclusion of individuals with comorbidities would have been both challenging and limited generalisability of findings.

We elected to include those with major depression where it was felt that HAND diagnosis was clear. Ideally, we would have reassessed following treatment but this was impossible within available resources. Moreover, depression may represent part of the neurobiological phenotype of HAND as well as a psychological response to HIV infection. Separation of these groups clinically would have been impossible and their exclusion unlikely to be representative.

The clinic was at times noisy and a suboptimal environment for neuropsychological testing. Nevertheless, we felt this was typical of other similar clinical settings.

Neuropsychological test performance is affected by many confounders. Our study controls were matched for age and educational level but not for other confounders including neurological or psychiatric impairments.

Neurological co-morbidity, such as VCI, was frequently identified. Detailed neurological evaluation was not the main aim of this study and without access to neuroimaging and specialist investigations it is likely many neurological disorders were missed. We therefore recorded comorbid neurological syndromes within case notes where clinically apparent and/or contributory to cognitive impairment. Similarly, HIV viral load testing was not locally-available and the effect of high viremia on cognitive performance could not be examined.

Childhood disadvantage is another confounding factor that may have affected cognitive performance in our study [60]. Although accurate retrospective measurement by self-report is challenging in later life, we could have measured well-recognised proxy markers such as head circumference and femur length particularly when comparing participant and control samples [61]. This is a limitation to the study. This will be an increasingly important area in future research.

Finally, for this clinic-based study we were only able to assess PLWH actually attending the HIV clinic, and not those previously lost to follow-up. Nevertheless, we felt they were likely to be typical of similar cohorts attending Government HIV services in Tanzania.

## Electronic supplementary material

Below is the link to the electronic supplementary material.

Supplementary file1 (DOCX 33 kb)
