# Supplementary material for: Screening for HIV-Associated Neurocognitive Disorder (HAND) in Adults Aged 50 and Over Attending a Government HIV Clinic in Kilimanjaro, Tanzania. Comparison of the International HIV Dementia Scale (IHDS) and IDEA Six Item Dementia Screen
Source: AIDS Behav. 2020 Sep 1;25(2):542–53. doi: 10.1007/s10461-020-02998-9 (PMC7846532; doi:10.1007/s10461-020-02998-9)
Supplement: Supplementary file 1 — Supplementary file1 (DOCX 33 kb) [file 10461_2020_2998_MOESM1_ESM.docx]

**Supplementary File**

**IDEA six-item cognitive screen**

**First attempt**: Now tell me all the words you can remember (tick on the grid the words remembered)

**Second attempt:** Now I will read out the words again, listen carefully and I will ask you to repeat as many as you can. Now tell me all the words you can remember (tick on the grid the words remembered)

**Third attempt:** Now I will read out the words one last time, listen carefully and I will ask you to repeat as many as you can. Now tell me all the words you can remember (tick on the grid the words remembered)

| Section A.3 | First attempt A.3.1 | Second attempt A.3.2 | Third attempt A.3.3 |
| --- | --- | --- | --- |
| Butter (siagi) |  |  |  |
| Arm (mkono) |  |  |  |
| Letter (barua) |  |  |  |
| Queen (malkia)) |  |  |  |
| Ticket (tikiti) |  |  |  |
| Grass (nyasi) |  |  |  |
| Corner (kona) |  |  |  |
| Stone (jiwe) |  |  |  |
| Book (kitabu) |  |  |  |
| Stick (fimbo) |  |  |  |

| 1 | I will tell you the name of something and I want you to describe what it is. **What is a bridge?** | **0** - incorrect **2** - correct (correct answer: something that goes across a river, canyon or road) | Score:____/2 |
| --- | --- | --- | --- |
| 2 | I want you to name as many **different** animals as you can in one minute.  Number of animals named: ____ | **0** - 0-3 animals named  **1** - 4-7 animals named  **2** - 8 or more animals named. | Score: ____/2 |
| 3 | Who is the chairman of your village? (Or street leader)) | **0** –incorrect **1** - correct | Score:____/1 |
| 4 | What day of the week is it? | **0** - incorrect **2** - correct | Score:____/2 |
| 5 | Can you tell me the ten words we learned earlier?  Try to remember as many as you can. | **0** no words remembered  **1** 1 word  **2** 2 words  **3** 3 words  **4** 4 words  **5** 5 or more words | Score:____/5 |
| 6 | Can you make the design shown below using these four matchsticks. I will show you once and then you have to copy exactly) (The examiner should make the design first using the matchsticks and specifically point out that the heads of the matchsticks all need to point the same way. Once the examiner has made the shape, collect up the matchsticks and place them in front of the person being interviewed) | Score **1** for each part of the design that is performed correctly  **1-**Middle two matchstick heads pointing same way  **1**-Outside two matchsticks pointing at an angle  **1-** Matchstick heads are orientated correctly | Score :____/3 |
| 7. | Allow the person to see the design as they make the shape*.* | Score as ABOVE ***(only if failed previously at item 6)*** | Score :____/3 |
|  | Best score from 6 or 7 | Use best score of 6 or 7 | Total:____ |
